# Empathic Responses for Pain in Facial Muscles Are Modulated by Actor’s Attractiveness and Gender, and Perspective Taken by Observer

**DOI:** 10.3389/fpsyg.2019.00624

**Published:** 2019-03-21

**Authors:** Kamila Jankowiak-Siuda, Anna Duszyk, Aleksandra Dopierała, Krzysztof Bujwid, Krystyna Rymarczyk, Anna Grabowska

**Affiliations:** Faculty of Psychology, Department of Experimental Neuropsychology, Institute of Cognitive and Behavioural Neuroscience, SWPS University of Social Sciences and Humanities, Warsaw, Poland

**Keywords:** empathy, pain, attractiveness, perspective taking, gender

## Abstract

Although empathy for pain is an often studied phenomenon, only few studies employing electromyography (EMG) have investigated either emotional responses to the pain of others or factors that modulate these responses. The present study investigated whether the sex and attractiveness of persons experiencing pain affected muscle activity associated with empathy for pain, the *corrugator supercili* (CS) and *orbicularis oculi* (OO) muscles, in male and female participants in two conditions: adopting a perspective of “the other” or “the self.” Fifty one participants (27 females) watched movies showing situations that included the expression of pain, with female and male and more and less attractive actors under both conditions, while the CS and OO EMG were recorded. Perspective did not affect CS muscle activity, but OO muscle activity tended to be higher in women than men under the imagine-self condition. CS muscle activity, but not OO muscle activity, was modulated by the actors’ gender and attractiveness. CS muscle activity was stronger in response to the pain of less attractive than more attractive actors, and to the pain of female actors compared to male actors. Moreover, a positive correlation was found between empathic concern, as a trait, and CS muscle activity, but only in the imagine-self condition.

## Introduction

One hundred and fifteen years ago, having described empathy as *Einfühlung* (“feeling into”), Theodor Lipps suggested that the perception of emotions of other persons triggers analogous emotions in the observer (Montag et al., 2008). The phenomenon assumes the existence of autonomic and somatic reactions in the observer leading to similar emotions, so-called “inner imitation.” Emotional mimicry can be considered an expression of imitation. This phenomenon is also an aspect of contemporary concepts of empathy, such as the perception-action model (PAM) of empathy (Preston and de Waal, 2002). PAM posits that the perception of the emotional state of others leads to the automatic activation of a representation of the same emotion in the observer, including autonomic and somatic reactions. Thus, PAM points to emotional mimicry as the most primal component of empathy (Hatfield et al., 1992; Preston and de Waal, 2002). This idea has received support from findings showing that the prevention of motor imitation of facial expressions makes recognizing emotions and affective sharing difficult (Sonnby-Borgström, 2002; Oberman et al., 2007; Dimberg et al., 2011). However, recent studies have revealed that facial mimicry is a more dynamic process than initially assumed (Sato et al., 2008; Rymarczyk et al., 2016a). Some studies indicate that facial mimicry depends on a number of modulating factors (Hess and Fischer, 2014; Seibt et al., 2015). The reaction of facial muscles to basic emotions depends on the sex or other characteristics of the observer. For example, the facial muscles have a noticeably greater reaction to basic emotions in highly empathic people (Dimberg et al., 2011; Balconi and Canavesio, 2016; Rymarczyk et al., 2016b). In addition, the reaction of the facial muscles is altered by levels of oxytocin (Korb et al., 2016). The type of the stimulus (dynamic vs. static) (Weyers et al., 2006; Sato et al., 2008; Rymarczyk et al., 2016a,b, 2018) also affects the reaction of the facial muscles to basic emotions.

Only a few studies using electromyography (EMG) have investigated emotional responses to the pain of others and the factors that modulate these reactions. Sun et al. (2015) showed that the activity of the *corrugator supercili* (CS) muscle increased in a group of research subjects who watched a short movie clip in which a person experienced pain (needle sticking), in comparison with a group who watched only part of the same movie (needle sticking the finger). Lamm et al. (2008) showed that the activity of the *orbicularis oculi* (OO) muscle was higher in study participants who watched a movie showing reactions to pain and who were instructed to imagine that they had experienced the pain (the “imagine-self” perspective), as compared to the “imagine-others” perspective (imagine what the person in the movie feels). In addition, Lepron et al. (2015) showed how a sense of responsibility for the suffering of another person affects emotional mimicry. Greater activity was observed in the OO and CS muscles and greater discomfort was reported by subjects who felt greater responsibility for other people’s pain, compared to subjects in the lesser responsibility condition.

In essence, two muscles (the CS and OO) may have special significance for the processing of empathy with pain. It should be noted that Lamm et al. (2008) and Sun et al. (2015) found positive correlations between the activity of the CS and OO muscles with scales measuring empathy, i.e., empathic concern (feeling compassion for others) and perspective taking (tendency to take another person’s perspective), from the Interpersonal Reactivity Index (IRI) (Davis, 1980).

Attractiveness is considered an important factor in social cognition for perceiving and empathizing with the pain of others (Jankowiak-Siuda et al., 2015). Research has shown that attractiveness has a particularly strong relationship with the reactions of the facial muscles of observers. For example, the attractiveness of a person’s face was found to be negatively correlated with the activation of the CS and the *legator labii superioris* (LLS) while viewing photographs of attractive and unattractive people (Principe and Langlois, 2011). Less attractive faces activated the LLS and CS more strongly. Neither the gender of the observer nor the gender of the person in the photograph were related to the activity of these facial muscles (Principe and Langlois, 2011). To date no relationship has been found between the reactions of the facial muscles of observers empathizing with a suffering person and the gender or attractiveness of the person suffering from pain. Therefore, the aim of the present study was to examine whether the gender or attractiveness of a person experiencing pain affects the activity of muscles associated with empathy for pain (the CS and OO) in male and female participants. In addition, the study measured the correlations of the electrical activity of the CS and OO facial muscles with the three empathy dimensions of the IRI (Davis, 1980): empathic concern, personal distress (feelings directed toward oneself), and perspective taking.

The perception of pain and empathy depends on adopting perspectives – whether we imagine how another person feels (“imagine-others”) or how we would feel (“imagine-self”) (Batson et al., 1997; Lamm et al., 2008). Imagining others evokes empathic concern, whereas imagining the self produces both empathic concern and personal distress; however, the latter is more in the way of egoistic negative emotions (Batson et al., 2003; Lamm et al., 2007). Thus, it is interesting to test whether perspective taking affects the activity of the CS and OO muscles when the attractiveness of the target varies. Based on available research findings we predict CS activity to be higher when observing unattractive women, no matter what perspective was adopted. However, we also predict OO activity to be higher for the imagine-self perspective than for the imagine-others perspective. Finally, we expect positive correlations between CS activity and empathic concern and between OO activity and perspective-taking.

The participants in the present study were shown dynamic pain stimuli in movies showing natural, everyday situations, in which they saw the whole body and the facial expressions of pain of actors (the targets) and the location of the injury. These stimuli were used because they have higher ecological validity than movies showing only the injured body part or expressions that mimic pain (Sun et al., 2015). Moreover, Sun et al. (2015) suggest that such situations are more likely to elicit empathy for pain. The present research measured relative changes in the activity of the CS and OO muscles during consecutive scenes. This enabled us to determine how the activity of the muscles changed in different scenes (showing the target’s neutral facial expression, the action leading to the pain, the actual pain stimulus, and the target’s facial expression of pain) depending on the sex and attractiveness of the target and the perspective taken by the observer.

## Materials and Methods

### Participants

Fifty one individuals participated in the study (women = 27; 20–39 years of age; *M* = 25.42, *SD* = 4.66). All the participants were right-handed and had normal or corrected to normal vision, and none of them suffered from neurological or psychiatric disorders. Financial compensation (about 5 Euros) was given to the participants. The project was approved by the Research Ethics Committee of the University of Social Sciences and Humanities in Warsaw, Poland. Informed, written consent was obtained from all of the participants.

### Stimuli

We used movies consisting of a series of approximately 6 s video-clips as stimuli to determine the effect of physical attractiveness on pain empathy. The movies showed targets in four pain-inducing situations: smashing their fingers, burning their lips, hitting their feet, and pricking their fingers. Four actors served as the targets in the movies: one man and one woman were assessed to be more attractive and one man and one woman were assessed to be less attractive. Each actor and actress portrayed each situation, making 16 scenes in total. Detailed descriptions regarding choosing the actors, assessing their attractiveness, and movie production are available in Jankowiak-Siuda et al. (2015). The selection of actors for their attractiveness included the following steps. First, two independent judges, both of whom were psychologists, selected 15 candidates (7 women, mean age = 20–35; and 8 men, mean age = 20–35) based on attributes of physical attractiveness; for example, facial symmetry and the shape and size of the nose (Perrett et al., 1998). Next, 347 persons, aged 20–40, evaluated the candidates’ attractiveness on a 5-point scale that ranged from 1 (completely unattractive) to 5 (very attractive). Based on the ratings, four actors with the following average attractiveness scores were selected: attractive = 4.15 (female *M* = 4.34, *SD* = 0.62; male *M* = 3.96, *SD* = 0.623) and less attractive = 1.65 (female *M* = 1.48, *SD* = 0.69; male *M* = 1.82, *SD* = 0.77). As the EMG signal has a very good time resolution, we reassembled all the video-clips in the same way, so that each video-clip scene in the movies had exactly the same duration. The scenes were shown in the following order:

• Scene 1 – actress’/actor’s face with a neutral expression – duration 520 ms
• Scene 2 – action leading to the pain (e.g., putting documents into a file drawer, in which the whole body is seen) – duration 1960 ms
• Scene 3 – actual pain stimulus (e.g., slamming a hand with a file drawer) – duration 240 ms
• Scene 4 – painful facial expression – duration 3000 ms

**FIGURE 1:**
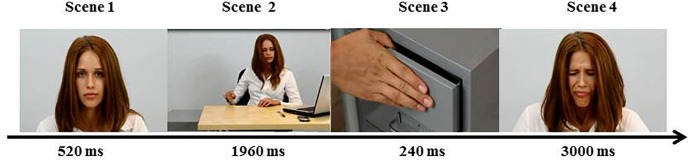
A sample of the video sequence. As an example, an attractive woman smashing her finger with the drawer is shown.

The course of an exemplar movie is depicted in Figure 1 below.

### EMG Data Acquisition

The level of muscle activity was measured with facial surface EMG. Data acquisition was performed using a TMSI Porti amplifier^1^ with a sampling frequency of 1024 Hz. It was connected to the computer via a USB interface using optical fiber. An area of the face was prepared before placing the electrodes, using conductive gel in order to reduce skin impedance. Two pairs of bipolar electrodes were placed above the CS and OO muscles, both of which were located on the right side of the face. Electrodes were placed according to the guidelines Fridlund and Caciopo, 1986. The ground electrode was placed on the chest near the breastbone. Dedicated software was used for data acquisition (SVAROG) and stimuli presentation (Psychopy). This software is available under the terms of the GPL license from http://psychopy.org and http://braintech.pl/svarog.

### Graphic Rating Scale (GRS)

A Graphic Rating Scale was used to evaluate the intensity of pain experienced by the actor in the movie, ranging from 0 = “no pain” to 10 = “the strongest pain one can imagine” (Haefeli and Elfering, 2006).

### Interpersonal Reactivity Index (IRI)

We used a Polish adaptation of the IRI (Davis, 1980) to measure empathy as a trait. The IRI consists of 28 questions that are answered using a 5-point scale that ranges from “I totally disagree” to “I strongly agree.” The Polish version the IRI (Kaźmierczak et al., 2007) consists of three scales: Empathic concern (EC), Personal distress (PD), and Perspective taking (PT) (after validation, the Fantasy scale was excluded from the Polish adaptation). The EC scale measures empathic concern for others, the PD scale measures feelings directed toward oneself (fear, distress, and discomfort), and the PT scale measures the tendency to take another person’s perspective.

### Procedure

The experiment was conducted in a darkened room with window curtains. A ceiling lamp was the only light source. The participants sat on a swivel chair half a meter from the center of the computer display. All the participants were informed that they would be watching short movies presenting situations involving pain reactions. They were expected to adopt two different perspectives: their own perspective (imagine-self condition) or the perspective of a third person (imagine-other condition) based on Lamm et al. (2008), in accordance with the instructions presented on the screen. In the imagine-self condition the instruction was “Imagine that you are in the place of the person and you are experiencing the pain;” in the imagine-other condition the instruction was “Imagine how the person feels while experiencing the pain.” The entire procedure was divided into two parts: a training session, in which a participant could become familiar with the procedure, and a main session. The training session consisted of three trials that showed pain scenes with different actors and situations than those in the main study. The aim of the training session was to enhance participants’ skills in perspective taking.

Sixteen video-clips were shown (described in the Stimuli section) twice during the main session: (a) in the imagine-self condition, and (b) in the imagine-other condition. Participants were randomly assigned to two groups: half of the participants started in the imagine-self condition, and the other half started in the imagine-other condition. Each trial began with a fixation cross for a variable interval of 3500 to 7500 ms, during which baseline muscle activity was established. Next, a movie was presented for 5720 s. The participant rated the intensity of pain shown by an actor on the GRS scale after each movie. The EMG signal was recorded from the CS and OO muscles during the entire experiment. Participants were asked to complete the Polish IRI after the session, to measure their level of trait empathy.

### EMG Data Analysis

The data was analyzed in MATLAB, using scripts written in-house. After visual inspection of the acquired signals, trials with artifacts (caused, for example, by frowning or yawning during the movies) were marked and excluded from further analysis. The resulting EMG signals were filtered with low-pass (Butterworth, cut-off frequency = 400 Hz), high-pass (Butterworth, cut-off frequency = 30 Hz) and notch (Butterworth, 49.9–50.1 Hz, 99–101 Hz, 149–151 Hz) filters. Afterward, trials related to movie scenes were cut into 8720 ms epochs: 2500 ms fixation point before the movie + 5720 ms movie presentation + 500 ms after the movie. A low frequency part of the absolute value of the phase-unlocked EMG response to a stimulus was obtained with a low-pass filter (Butterworth, cut-off frequency = 20 Hz) in order to get the signal envelope. A signal baseline value was calculated as the average of the samples from the fixation period (2000 ms before the movie in each trial).

In order to compare particular trials, the relative increase/decrease of the amplitude was calculated by dividing every signal sample by the baseline value. These prepared epochs were grouped according to the following criteria: the actor’s gender, the participant’s gender, and the actor’s attractiveness in each condition (imagine-self and imagine-other). Next, the mean value of the signal in the time domain was calculated for each scene. After that, all these values were averaged in the trial domain, producing the mean value for each scene; statistical tests were used to compare the scenes.

### Statistical Analyses

Such prepared EMG indicators were compared with behavioral scores using ANOVA and *post hoc* tests. A repeated-measures analysis of variance (ANOVA) was performed, with a single between-subjects factor and the following within-subjects factors: (1) the actor’s gender (female vs. male), (2) the actor’s attractiveness (low vs. high), (3) perspective-taking condition (imagine-self vs. imagine-other), and (4) scenes from the movies (actress’s/actor’s neutral face, action leading to pain, actual pain stimulus, and painful facial expression). The between-subjects factor was the subject’s gender (male vs. female). The analysis was performed separately for the CS and OO muscles and the GRS score (the GRS analysis was conducted without the scene from the movie as a factor). *Post hoc* analyses were done for every possible comparison using Sidak’s correction and paired *t*-test with the value of Cohen’s d effect size calculated when appropriate.

Pearson correlation coefficients were calculated for the mean signal value separately for each scene separately for the CS and OO muscles and three scales of IRI: EC, PD, PT with the FDR correction for multiple comparisons. All analyses were conducted using SPSS version 25.

## Results

### Analysis of the Pain Intensity Ratings (GRS Score)

The analysis revealed a main effect of perspective taking, *F*(1, 49) = 7.867, *p* < 0.05, η^2^ = 0.138, attractiveness of the actor, *F*(1, 49) = 62. 365, *p* < 0.001, η^2^ = 0.560, and gender of the actor, *F*(1, 49) = 95.842, *p* < 0.001, η^2^ = 0.662. Participants rated the pain as being more intense when adopting the other’s perspective (*M* = 4.62) than when adopting the self perspective (*M* = 4.39). Moreover, participants rated the intensity of the pain expressed by less attractive actors (*M* = 4.97) higher than they rated the intensity of pain expressed by attractive actors (*M* = 4.47), *p* < 0.001. In addition, participants rated the pain intensity of the female actors higher (*M* = 5.07) than the pain intensity of the male actors (*M* = 4.37, *p* < 0.001). There was also a significant interaction between the gender and attractiveness of the actors, *F*(1, 49) = 23.022, *p* < 0.001, η^2^ = 0.320. To explain the interaction effect, an analysis of the main effect of the gender of the actors (with Sidak’s correction) was conducted separately for the less and more attractive actors, and the analysis of the main effect of the attractiveness of the actors was conducted separately for female and male actors. As may be seen, the Sidak’s *post hoc* analysis could not explain the interaction effect. Thus, paired *t*-test was conducted and the value of Cohen’s d effect size was calculated. Although each of the *t*-tests brought significant results [*t*(50) = 10.203, *p* < 0.001; *t*(50) = 5.924, *p* < 0.001; *t*(50) = 8.036, *p* < 0.001; *t*(50) = 4.363, *p* < 0.001, for less attractive men vs. less attractive women, attractive women vs. attractive men, attractive women vs. less attractive women, and attractive men vs. less attractive men, respectively], the largest Cohen’s d value was revealed when less attractive men and less attractive women means of the GRS scores were compared (*d* = 1.43). The effect size can be interpreted as very large. The other values were as follows: *d* = 0.83 (large effect size), *d* = 1.13 (large effect size), and *d* = 0.61 (medium effect size), for attractive women vs. attractive men, attractive women vs. less attractive women, and attractive men vs. less attractive men, respectively.

**FIGURE 2:**
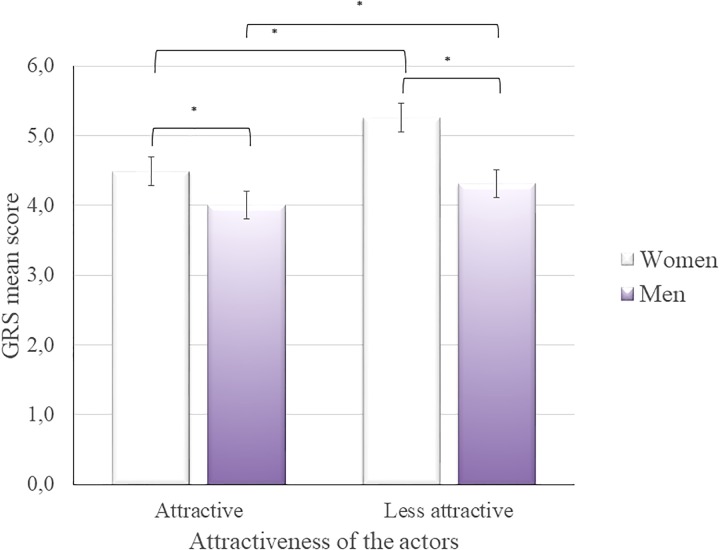
Mean (±SEM) evaluations of observed pain intensity (GRS) by gender and attractiveness of the actors. ^∗^*p* < 0.001.

The pain expressed by less attractive women was rated as more intense (*M* = 5.45) than that expressed by more attractive women (*M* = 4.69, *p* < 0.001). In addition, the pain expressed by less attractive men was rated as more intense (*M* = 4.49) than the pain expressed by more attractive men (*M* = 4.24, *p* < 0.001). The results are presented at Figure 2. There was no effect of the gender of the observers on the pain intensity ratings (see Supplementary Material for details).

### Analysis of the Relative Change in the EMG Amplitude of the CS Muscle

There was no effect of perspective taking on the relative change in the EMG amplitude of the CS muscle: i.e., the relative change was similar in both the imagine-self and imagine-other conditions. However, there was a main effect of the attractiveness of the actress/actor, *F*(1, 48) = 9.24, *p* = 0.004, η^2^ = 0.161, and a main effect of the scene, *F*(3, 144) = 9.91, *p* < 0.001, η^2^ = 0.171.

**FIGURE 3:**
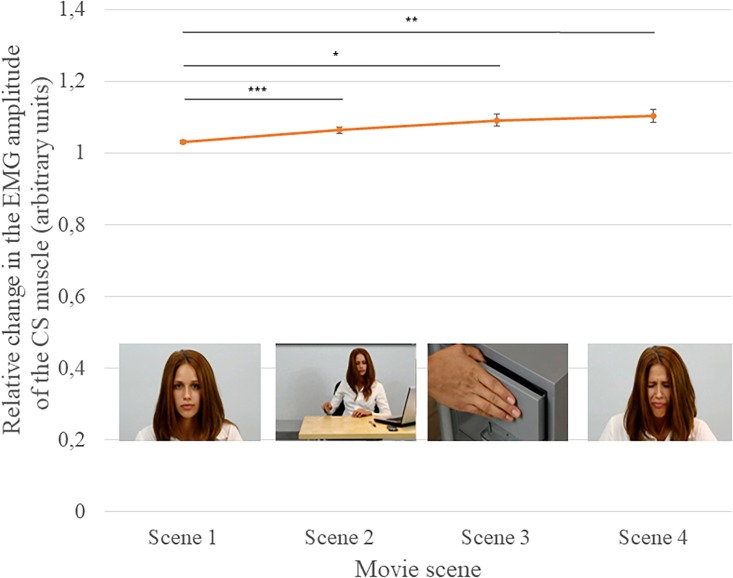
Mean (±SEM) relative change in the EMG amplitude of the CS muscle in each of the four scenes; ^∗^*p* = 0.002; ^∗∗^*p* = 0.001; and ^∗∗∗^*p* < 0.001.

Participants showed a greater change in the EMG amplitude of the CS muscle when observing pain expressed by less attractive persons (*M* = 1.082) than when observing pain expressed by more attractive persons (*M* = 1.063). The relative change in EMG amplitude increased from scene to scene (Scene 1 *M* = 1.032; Scene 2 *M* = 1.064, Scene 3 *M* = 1.092, and Scene 4 *M* = 1.103). Contrast analysis revealed that the differences were significant between Scene 1 (neutral facial expression) and all three other scenes: Scene 1 vs. Scene 2 (*p* < 0.001); Scene 1 vs. Scene 3 (*p* = 0.002), and Scene 1 vs. Scene 4 (*p* = 0.001) (see Figure 3).

**FIGURE 4:**
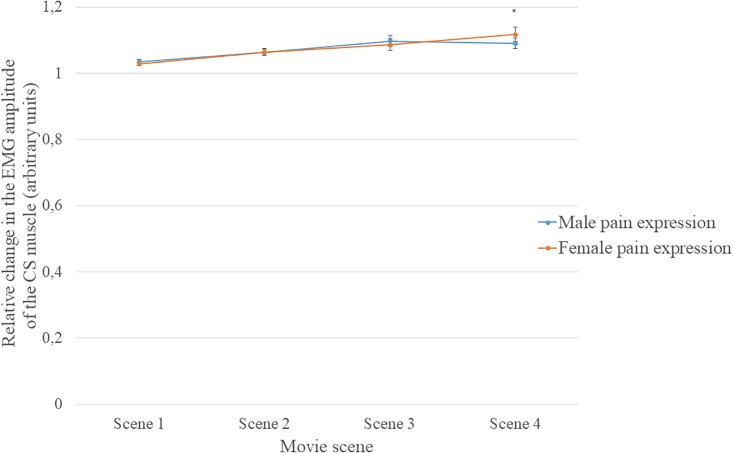
Mean (±SEM) relative change in the EMG amplitude of the CS muscle when participants were watching male and female actors in each of the four scenes; ^∗^*p* < 0.05.

There was also a statistically significant interaction between the gender of the actor and the scene, [*F*(3, 144) = 4.40; *p* = 0.005; η^2^ = 0.084]. Analysis of the simple main effects of the gender of the actor (using Sidak’s correction) for each scene revealed a significant difference in Scene 4. Participants exhibited a larger relative change in EMG amplitude when watching pain expressed by women (*M* = 1.117) than by men (*M* = 1.090, *p* < 0.05). Differences in the relative EMG amplitude by gender of the actors were not statistically significant in Scenes 1, 2, and 3 (see Figure 4).

No other effects or comparisons were statistically significant (see Supplementary Material for details).

### Analysis of the Relative Change in the EMG Amplitude of the OO Muscle

**FIGURE 5:**
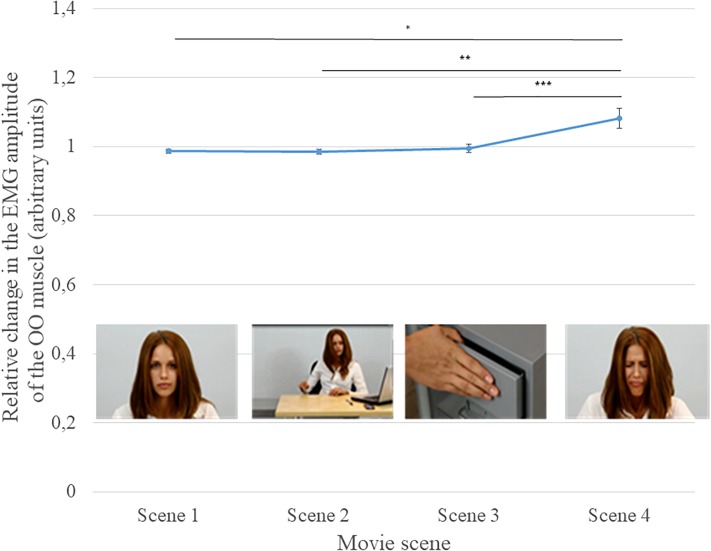
Mean (±SEM) relative change in the EMG amplitude of the OO muscle in each of the four scenes; ^∗^*p* < 0.05; ^∗∗^*p* < 0.01; and ^∗∗∗^*p* < 0.001.

Analysis of the relative change in the EMG amplitude of the OO muscle revealed a significant main effect of the scene, *F*(3, 135) = 11.267, *p* < 0.001, η^2^ = 0.20. Compared to Scene 4, which showed the actors’ facial expression of pain (Mean amplitude change = 1.083), the participants showed smaller changes in EMG amplitude in Scene 1 (showing the actor’s neutral face: *M* = 0.987, *p* = 0.01), Scene 2 (the action leading to the pain: *M* = 0.985, *p* = 0.005), and Scene 3 (the actual pain stimulus: *M* = 0.994, *p* = 0.003) (see Figure 5).

Moreover, the analysis revealed a statistical tendency for the interaction between perspective taking and the gender of the participant, *F*(1, 45) = 3.29, *p* = 0.077, η^2^ = 0.068. The relative change in the EMG amplitude of the OO muscle was somewhat larger in the imagine-self condition than in the imagine-other condition for female participants (*M* = 1.026, *p* = 0.170). No other effects or comparisons were statistically significant (see Supplementary Material for details).

### Relationship Between Empathy Measures and the Relative Change in EMG Amplitude of the CS and OO Muscles

Pearson’s correlation coefficient (*r*) was used to measure the association between the participants’ scores on the IRI subscales (Empathic Concern, Personal Distress, and Perspective Taking) and the relative change in the EMG amplitudes of the CS and OO muscles, during each of the four movie scenes, in the imagine-self and imagine-other conditions.

There was a significant positive correlation [*r*(46) = 0.42; *p* = 0.03] between Empathic Concern and the relative change in the EMG amplitude of the CS muscle for the Scene 4 – painful facial expression in the imagine-self condition. All other correlations were not significant (see Supplementary Material for details).

## Discussion

The main objective of this study was to determine how the attractiveness and gender of a person experiencing pain affects the activity of the CS muscle and the OO muscle in male and female observers adopting the perspective of the other (the observer imagining how the person in the movie feels) and the perspective of the self (the observer imagining how s/he would feel being in the place of the person in the movie). We hypothesized that CS activity will not change no matter what perspective is adopted but OO activity will be higher for the imagine-self perspective than for the imagine-others perspective. Moreover, we expected the CS activity to be higher when observing less attractive women than any other actors. We expected to obtain positive correlations between CS activity and empathic concern and between OO activity and perspective-taking. The study did not find an effect of perspective taking (other and self) on the activity of the CS muscle. Interestingly, however, the activity of the OO muscle showed a trend in the interaction between gender of the observer and perspective taking: when the imagine-self perspective was adopted, OO muscle activity was stronger in women than in men. This result is more specific than that reported by Lamm et al. (2008), who reported generally stronger OO muscle activity in the imagine-self than in the imagine-other condition. It should be noted that women, compared to men, exhibit enhanced activity of the mirror neuron system (MNS) (*right inferior frontal cortex* and the *superior temporal sulcus*) during imagine-self perspective taking (Schulte-Ruther et al., 2008; Marzoli et al., 2011). In addition, similar enhanced MNS activity in women, in comparison to men, was found when participants observed pain stimuli applied to moving hands and legs (Cheng et al., 2007, 2008; Marzoli et al., 2011). In essence, this may imply that motor imagination in women is very important for empathizing with others and for faster emotional mimicry (Marzoli et al., 2011). Moreover females seem more reactive in facial muscle reactivity than are males (Dimberg and Lundquist, 1990). Our findings are in line with a number of studies showing that the activation of empathy in women is more closely related to affective sharing than it is in men (Hoffman, 1977; Eisenberg and Lennon, 1983; Dimberg and Lundquist, 1990; Sonnby-Borgström et al., 2008, Marzoli et al., 2011).

The results of the behavioral aspects of the study revealed differences in the evaluation of observed pain intensity related to the perspective adopted. The participants rated the pain as stronger when adopting the “other” perspective than the “self” perspective. Previous research reported that perspective either had no effect on the evaluation of pain intensity (Lamm et al., 2008) or that pain was evaluated as being stronger in the “self” condition (Jackson et al., 2006). This discrepancy may be related to the different stimuli used in the studies, which may be relevant for the perspective adopted. Lamm et al. (2008) showed faces expressing pain reactions, whereas Jackson et al. (2006) used pictures showing pain; the movies used in the present study showed the entire scenario leading up to the pain.

The analysis of the effects of the attractiveness of the actors revealed significant results only for the CS muscle. It revealed that the change in the activity of the CS muscle was greater when watching the experience of pain of a less attractive actor than the pain of a more attractive one. This finding agrees with the results of previous research (Principe and Langlois, 2011), in which stronger muscle activity was recorded during the presentation of neutral faces rated as unattractive. The activity of the CS muscle, as previously shown, is particularly strong when negative emotions are felt, such as anger, sadness, or disgust (Dimberg and Petterson, 2000; Hess and Blairy, 2001; Hess and Fischer, 2014) and in response to pain (Lepron et al., 2015; Sun et al., 2015). Thus, the large change in the activity of the CS muscle when watching the expression of pain by less attractive people may be related to stronger negative affect upon seeing their pain, in comparison to watching the expression of pain by more attractive people. Low attractiveness is related to such attributes as poor health, lower immunity, and fewer resources to cope with pain (Zebrowitz et al., 2002; Weeden and Sabini, 2005; Mobius and Rosenblat, 2006), and as a result, negative affect may increase. However, such attributions may also favor greater empathy for the pain of such people. Indeed, the positive correlation between the activity of the CS muscle and the level of empathic concern revealed in the present research and by Sun et al. (2015) suggest that this muscle may be regarded as some kind of the marker for empathic concern, but only when the stimuli show the entire situation leading to pain.

In addition, the increase in CS muscle activity was observed in consecutive scenes of the movie as the possibility of the aversive reaction in response to the stimuli increased: from the presentation of the neutral face of the actor (Scene 1), to the context of the pain stimulus (Scene 2), to the application of the pain stimulus (Scene 3), and finally, the painful expression on the actor’s face (Scene 4). These results agree with the results of studies demonstrating an increase in CS activity when the strength of an unpleasant experience is increased (Magnee et al., 2007; Lamm et al., 2008).

It is noteworthy that the reaction of the CS muscle depended not only on the movie scene (which was strongest when the expression of pain was shown), but also on the gender of the actor. In the 4th scene of the movie, when the pain expression on the actor’s face was visible, the activity of the CS muscle was greater for female than for male actors. The result seems to support the idea that the activity of the CS muscle is closely related to the negative affect felt in response to a number of factors (Dimberg and Petterson, 2000; Hess and Blairy, 2001; Hess and Fischer, 2014). On one hand, the muscle responds strongly at the sight of unattractive faces that could trigger negative affect (Principe and Langlois, 2011). On the other hand, the muscle responds more strongly to the observation of pain experienced by women, which also might lead to stronger negative affect. The stronger activity of the CS muscle in the 4th scene, in response to the pain visible on the faces of the women than to the pain visible on the faces of the men, accords with the results of studies reporting that women’s pain is rated as more intense than men’s pain (Hadjistavropoulos et al., 1990, 1996; Robinson and Wise, 2004; Gleichgerrcht and Decety, 2014; Sadeghiyeh et al., 2017). Moreover, this could be a consequence of the social perception of women as more sensitive to pain, fragile, and requiring more care and support (Hall and Schmid Mast, 2008; Gleichgerrcht and Decety, 2014). The empathic concern for a person in pain should be particularly strong in the scene where the pain is visible on the women’s faces. It should be noted that our behavioral data revealed the highest ratings of pain intensity were when the pain was visible on the face of an unattractive woman, compared to the three other actors. This suggests that the evaluation of pain intensity may affect the activity of facial muscles, especially the CS muscle. In addition, the results indicate that the activity of the CS muscle is related to empathy. It should be borne in mind that the positive correlation between the empathic concern subscale of the IRI and activity of the CS muscle was found only when participants were watching the movies while adopting the imagine-self perspective. The empathic concern subscale measures the tendency to concentrate on the feelings of another person and the level of concern toward another person’s sufferings and failures. Interestingly, in the present research, imagining what another person feels by putting oneself in the place of the other revealed a strong relationship between concentrating on one’s own feelings and self-concern. This may be related to Western, individualistic culture, which strongly emphasizes independence and the focus on oneself, which implies a tendency to concentrate on one’s own feelings (Cheon et al., 2013). The results of Sun et al. (2015) study revealed, for the first time, the relationship between CS muscle activity and empathic concern in an Asian sample. Although the effect of perspective taking on the activity of facial muscles was not examined by Sun et al. (2015), the collectivist Asian culture is typically characterized by a strong focus on others and its emphasis on interdependence. Thus, perhaps, the relationship between empathic concern and facial muscle activity depends on perspective-taking, and this can be modified by the general cultural context. The relationship between culture and facial muscle activity in response to the pain felt by women and men requires further research, conducted using the same paradigms and the same stimuli.

Despite the foregoing relationship, it is interesting to note that there was no correlation between the activity of the OO muscle and dispositional empathy. Lamm et al. (2008) research found a positive correlation between the activity of the OO muscle and the IRI’s empathy subscale of perspective taking. Perhaps this was an effect of the specific stimuli used, but one cannot rule out the hypothesis that the activity of the OO muscle is related to the initiation of the empathic response and emotional contagion, rather than to being empathic *per se*. Other research has revealed that the activity of the OO muscle when one experiences pain is linked to the facial expression of the emotion (Wolf et al., 2005). In line with this reasoning, the present results reveal that OO muscle activity was strongest (significantly higher than in other three scenes) in the 4th and final scene of the movie when the expression of pain was visible on the face of the actors. The activity of the OO muscle was also differentiated in the Lepron et al. (2015) study, in which the level of responsibility for the pain of another person was manipulated. Increased muscle activity was observed when participants felt responsible for the pain of the other person. The participants in that study were convinced that they applied the painful stimuli to the person shown on the screen when they decided how strong the painful stimuli were. Thus, OO muscle activity may be related more to the intensity of the emotions one feels than to the valence of the emotion (Messinger et al., 2012).

The results of the present study and their interpretation certainly have some limitations, as the lack of a control condition which could strengthen the assumption that the EMG results reflect empathy, rather than some other effect. However, we administered the same pain stimuli (the same video scenes) as in an earlier study (Jankowiak-Siuda et al., 2015), in which we were able to show that watching the videos activated pain empathy brain areas. This response is in agreement with the assumption that watching the facial pain expression in others leads to empathic response in the observer (De Vignemont and Singer, 2006; Singer and Lamm, 2009) It should be noted, that the procedure itself (a perspective-taking task) was shown to elicit empathic response in the subjects (Batson et al., 1997; Jackson et al., 2006; Lamm et al., 2008). Moreover, our data revealed statistically significant positive moderate correlation between the CS muscle activity and the Empathic Concern scale of the IRI. Despite that, it certainly would be valuable to analyze changes in muscle activity while watching movies that do not show the application of pain stimuli or the expressions of pain.

Other limitation is the absences of ratings for attractiveness by this particular cohort of participants. One should notice, however, that we used attractiveness ratings carefully collected and verified in other studies (Jankowiak-Siuda et al., 2015).

One may also hesitate, if the participants did indeed adopt different perspectives during the task. One should consider, however, that although we did not observe a main effect of perspective taking (as Lamm et al., 2008), the interaction between the gender of the observer and perspective taking was close to significance for the activity of the OO muscle. Moreover, participants rated the pain as more intense when they adopted the perspective of the other than in those instances when they adopted the self perspective. Finally, we followed the perspective-taking procedure exactly as described by Lamm et al. (2008). It included a training session, designed to strengthen the subjects’ skills in performing the perspective-taking task. On the other hand, it would be interesting to see how the activity of the muscles change in a purely perceptual condition, without adopting any perspective. In summary, although empathizing with the pain of another person appears to take place at some “inner imitation” level involving facial muscle activity, the results support the idea that this process is not automatic (Rymarczyk et al., 2016a,b; Sato et al., 2008), but controlled by a “top-down” path (Lamm et al., 2008). We were able to reveal several main and interaction effects on facial muscle activity, especially CS muscle activity, by using and manipulating several variables simultaneously, including the attractiveness and gender of the actors, the gender of the participants, taking different perspectives (self vs. other), and using different scenes from the pain movies. First of all, the greatest change in CS muscle activity was observed during the 4th scene of the movie, when the expression of pain was visible on the face of the actors/actresses, and second, when the pain was visible on the face of an unattractive actress. Moreover, a positive correlation was found between CS muscle activity and empathic concern in the self-perspective condition, which was largest for the 4th scene of the movie, when the facial expression of pain was visible. This may support the assumption that mimicry is a primal empathic response and that imagining what one would feel being in the place of a suffering person is an important stage leading not only to personal distress, but to empathic concern. This would seem to be in agreement with Batson et al. (2003), who suggested that an imagine-self perspective can elicit personal distress as well as empathic concern. This issue requires further research to continue to examine whether and when the change in muscle activity occurs at the sight of pain, controlling for individual differences in level of empathy.

## Ethics Statement

This study was carried out in accordance with the recommendations of Research Ethics Committee of the SWPS University of Social Sciences and Humanities in Warsaw, Poland with written informed consent from all subjects. All subjects gave written informed consent in accordance with the Declaration of Helsinki. The protocol was approved by the Research Ethics Committee of the SWPS University of Social Sciences and Humanities.

## Author Contributions

KJ-S and AnD created and designed the experiments and analyzed the data. KJ-S, AnD, AlD, and KB performed the experiments. KJ-S, KR, AlD, and KB contributed to materials. KJ-S, AnD, AlD, KR, and AG wrote the manuscript.

## Conflict of Interest Statement

The authors declare that the research was conducted in the absence of any commercial or financial relationships that could be construed as a potential conflict of interest.

## Footnotes

1 http://tmsi.com
