# Supplementary material for: Empathic Responses for Pain in Facial Muscles Are Modulated by Actor’s Attractiveness and Gender, and Perspective Taken by Observer
Source: Front Psychol. 2019 Mar 21;10:624. doi: 10.3389/fpsyg.2019.00624 (PMC6437081; doi:10.3389/fpsyg.2019.00624)
Supplement: Supplementary file 1 [file Table_1.docx]

Supplementary material

**Analysis of the Pain Intensity Ratings (GRS Score)**

The following effects were not significant: main effect of sex of the observer (F(1, 49)=2.54; p=.118), interaction between perspective taking and the sex of the observer (F(1, 49)=2.061; p=.158), interaction between the sex of the actor and of the observer (F(1, 49)=0.653; p=.423); interaction between the attractiveness of the actor and sex of the observer (F(1, 49)=1.261; p=.267), interaction between perspective taking and the sex of the actor (F(1, 49)=0.221; p=.640), interaction between perspective taking, the sex of the actor, and the sex of the observer (F(1, 49)=2.002; p=.163), interaction between perspective taking and the attractiveness of the actor (F(1, 49)=0.554; p=.460), interaction between perspective taking, the attractiveness of the actor and the sex of the observer (F(1, 49)=0.270; p=.606), interaction between the sex and the attractiveness of the actor, and the sex of the observer (F(1, 49)=0.166; p=.685), interaction between perspective taking, the attractiveness and the sex of the actor (F(1, 49)=1.146; p=.290). There was also a statistically significant fourth level interaction between perspective taking, the attractiveness and the sex of the actor, and the sex of the observer (F(1, 49)=5.546; p=.023; η^2^=.102). The interaction was not analyzed in depth, because of the questionable interpretation due to the complexity.

**Analysis of the Relative Change in the EMG Amplitude of the CS Muscle**

The following effects were not significant: main effect of the sex of the observer (F(1, 48)=1.149; p=.289), main effect of perspective taking (F(1, 48)=2.782; p=.102), interaction between perspective taking and the sex of the observer (F(1, 48)=0.424; p=.518), main effect of the sex of the actor (F(1, 48)=0.177; p=.676), interaction between the sex of the actor and observer (F(1, 48)=0.172; p=.680), interaction between the attractiveness of the actor and the sex of the observer (F(1, 48)=1.491; p=.228); interaction between the scene and the sex of the observer (F(3, 144)=0.487; p=.692); interaction between perspective taking and the sex of the actor (F(1, 48)=1.070; p=.306), interaction between perspective taking, and sex of the actor and of the observer (F(1, 48)=0.048; p=.828), interaction between perspective taking and the attractiveness of the actor (F(1, 48)=1.063; p=.308), interaction between perspective taking, the attractiveness of the actor and the sex of the observer (F(1, 48)=0.017; p=.898), interaction between the sex and the attractiveness of the actor (F(1, 48)=0.023; p=.880), interaction between the sex and the attractiveness of the actor, and the sex of the observer (F(1, 48)=1.50; p=.227), interaction between perspective taking, and the attractiveness and the sex of the actor (F(1, 48)=0.01; p=.919), interaction between perspective taking, the attractiveness and the sex of the actor, and the sex of the observer (F(1, 48)=0.000; p=.985), interaction between perspective taking and the scene (F(3, 144)=0.194; p=.901), interaction between perspective taking, the scene and the sex of the observer (F(3, 144)=0.287; p=.835), interaction between the sex of the actor, the scene, and the sex of the observer (F(3, 144)=0.299; p=.826), interaction between perspective taking, the sex of the actor, and the scene (F(3, 144)=1.514; p=.213), interaction between perspective taking, the sex of the actor, the sex of the observer, and the scene (F(3, 144)=1.222; p=.304), interaction between the attractiveness of the actor and the scene (F(3, 144)=0.456; p=.714), interaction between the attractiveness of the actor, the scene, and the sex of the observer (F(3, 144)=1.522; p=.211), interaction between perspective taking, the attractiveness of the actor and the scene (F(3, 144)=0.890; p=.966), interaction between perspective taking, the attractiveness of the actor, the scene, and the sex of the observer (F(3, 144)=1.000; p=.395), interaction between the sex and the attractiveness of the actor, and the scene (F(3, 144)=0.148; p=.931), interaction between the sex and the attractiveness of the actor, the scene, and the sex of the observer (F(3, 144)=1.920; p=.129), interaction between perspective taking, the sex and the attractiveness of the actor, and the scene (F(3, 144)=2.103; p=.102), interaction between perspective taking, the sex and the attractiveness of the actor, the scene, and the sex of the observer (F(3, 144)=0.258; p=.856).

**Analysis of the Relative Change in the EMG Amplitude of the OO Muscle**

The following effects were not significant: main effect of the sex of the observer (F(1, 45)=0.152; p=.699), main effect of perspective taking (F(1, 45)=0.019; p=.890), main effect of the sex of the actor (F(1, 45)=1.358; p=.250), interaction between the sex of the actor and of the observer (F(1, 45)=0.023; p=.880), main effect of the attractiveness of the actor (F(1, 45)=1.262; p=.267), interaction between the attractiveness of the actor and the sex of the observer (F(1, 45)=0.261; p=.612), interaction between the scene and the sex of the observer (F(3, 135)=0.331; p=.803); interaction between perspective taking and the sex of the actor (F(1, 45)=0.121; p=.730), interaction between the sex of the actor and of the observer (F(1, 45)=1.004; p=.322), interaction between perspective taking and the attractiveness of the actor (F(1, 45)=0.232; p=.633), interaction between perspective taking, the attractiveness of the actor and the sex of the observer (F(1, 45)=0.274; p=.604), interaction between the sex and the attractiveness of the actor (F(1, 45)=0.183; p=.671), interaction between the sex and the attractiveness of the actor, and the sex of the observer (F(1, 45)=0.112; p=.740), interaction between perspective taking, and the attractiveness and the sex of the actor (F(1, 45)=0.061; p=.807), interaction between perspective taking, the attractiveness and the sex of the actor, and the sex of the observer (F(1, 45)=1.181; p=.283), interaction between perspective taking, and the scene (F(3, 135)=0.643; p=.589), interaction between perspective taking, the scene, and the sex of the observer (F(3, 135)=0.461; p=.710), interaction between the sex of the actor and the scene (F(3, 135)=0.400; p=.753), interaction between the sex of the actor, the scene, and the sex of the observer (F(3, 135)=1.335; p=.266), interaction between perspective taking, the sex of the actor and the scene (F(3, 135)=0.283; p=.837), interaction between perspective taking, sex of the actor and of the observer, and the scene (F(3, 135)=1.801; p=.150), interaction between the attractiveness of the actor and the scene (F(3, 135)=1.024; p=.384), interaction between the attractiveness of the actor, the scene, and the sex of the observer (F(3, 135)=1.381; p=.251), interaction between perspective taking, the attractiveness of the actor, and the scene (F(3, 135)=0.169; p=.917), interaction between perspective taking, the attractiveness of the actor, the scene, and the sex of the observer (F(3, 135)=0.141; p=.935), interaction between the sex and the attractiveness of the actor, and the scene (F(3, 135)=0.392; p=.759), interaction between the sex and the attractiveness of the actor, the scene, and the sex of the observer (F(3, 135)=1.679; p=.174), interaction between perspective taking, the sex and the attractiveness of the actor, and the scene (F(3, 135)=1.791; p=.152), interaction between perspective taking, the sex and the attractiveness of the actor, the scene, and the sex of the observer (F(3, 135)=0.538; p=.657).

Table 1
Correlation of the IRI Scales with the Relative Change in EMG Amplitude of the CS Muscle for Each of the Four Movie Scenes in the Imagine-Self and Imagine-Other Conditions

|  | Scene 1 Other | Scene 2 Other | Scene 3 Other | Scene 4 Other | Scene 1 Self | Scene 2 Self | Scene 3 Self | Scene 4 Self |
| --- | --- | --- | --- | --- | --- | --- | --- | --- |
| EC | .149 | .212 | .133 | .062 | .334^a^ | .361^a^ | .298^a^ | **.418^b^*** |
| PD | -.097 | .018 | -.179 | .261 | .123 | -.103 | -.178 | .237 |
| PT | .067 | .046 | .059 | -.164 | .301^a^ | .154 | .198 | -.040 |

*Note*. CS = *corrugator supercilii*; EMG = Electromyography; EC = Emphatic Concern; PD = Personal Distress, PT = Perspective Taking. All df's for r-Pearson values are 46.; ^a^ *p* < .05; ^b^ *p* < .01; with FDR correction; **p*=.03.

Table 2
Correlation of the IRI Scales with the Relative Change in EMG Amplitude of the OO Muscle for Each of the Four Movie Scenes in the Imagine-Self and Imagine-Other Conditions

|  | Scene 1 Other | Scene 2 Other | Scene 3 Other | Scene 4 Other | Scene 1 Self | Scene 2 Self | Scene 3 Self | Scene 4 Self |
| --- | --- | --- | --- | --- | --- | --- | --- | --- |
| EC | -.007 | -.119 | -.075 | .193 | .213 | -.024 | -.074 | -.129 |
| PD | .089 | .106 | -.188 | -.020 | .202 | .230 | .148 | .238 |
| PT | -.020 | -.229 | -.237 | -.016 | -.114 | -.103 | -.113 | -.074 |

*Note*. OO = *orbicularis oculi*; EMG = Electromyography; EC = Emphatic Concern; PD = Personal Distress; PT = Perspective Taking. All df's for r-Pearson values are 44.
